# Editorial: Unraveling the complex interplay between aging, immunity, metabolites, and metabolism in endocrine-related cancers

**DOI:** 10.3389/fendo.2024.1527867

**Published:** 2024-12-02

**Authors:** Guang-Liang Chen, Yubin Luo, Yuwei Zhang, Chao Liu

**Affiliations:** ^1^ Shanghai Cancer Center, Fudan University, Shanghai, China; ^2^ West China Hospital, Sichuan University, Chengdu, China; ^3^ Harvard Medical School, Boston, MA, United States

**Keywords:** endocrine-related cancers, aging, metabolic, breast cancer, immune, Mendelian randomization

Endocrine-related cancers span a diverse range of malignancies affecting organs such as the breast, thyroid, and adrenal cortex, each profoundly influenced by aging, metabolic reprogramming, and shifts in immune function. This editorial examines the contributions within this Research Topic, focusing on studies that explore the intersections of aging, immune modulation, and metabolic pathways and their roles in cancer progression and treatment outcomes.

A notable study by Feng et al. on metabolic reprogramming in gastric cancer introduces a robust metabolic model that categorizes patients based on metabolic activity, uncovering critical links to prognosis. This approach to metabolic profiling highlights the value of integrating metabolic states into tailored treatment strategies, advancing personalized care for gastric cancer patients.

Several studies in this Research Topic investigate HER2 expression in breast cancer, led by Professor Biyun Wang’s research group at the Fudan University Shanghai Cancer Center, a prominent center for breast cancer research in China. These studies collectively underscore the complexity of treatment strategies for HER2-targeted therapies. For instance, the work by You et al. on the clinicopathological characteristics and outcomes of HER2-low metastatic breast cancer demonstrates that HER2 expression varies significantly between primary and metastatic lesions, impacting treatment responses and underscoring the need for continuous HER2 monitoring to optimize therapy. Complementing this, a comparative study of pyrotinib versus trastuzumab in HER2+ metastatic breast cancer highlights the nuanced decision-making required in HER2-directed treatments (You et al.). Adding further depth to HER2 research, Hu et al.‘s pooled analysis of HER2-low, hormone receptor-negative metastatic breast cancer reveals distinct clinical characteristics in this subgroup. Notably, HER2-low patients are generally older and predominantly postmenopausal, yet they show comparable outcomes to HER2-0 patients when receiving platinum-based chemotherapy. These findings emphasize the clinical relevance of HER2-low as an actionable biomarker and underscore the need for further investigation into its prognostic significance. In a study from Prof. San-Gang Wu’s team at Xiamen Cancer Center, Liu et al. examine age-specific metastatic patterns in breast cancer, discovering a notable trend: older patients exhibit a higher tendency for lung rather than liver metastases and generally face poorer survival outcomes. This finding underscores the critical impact of aging on metastatic behavior in breast cancer, highlighting the need for tailored treatment strategies that address the unique challenges faced by older patients.

Mendelian randomization has emerged as a powerful tool for analyzing the interplay between different diseases, gaining traction in recent years for its potential to reveal causal relationships. This Research Topic includes several notable studies employing this approach. For example, Li et al. investigate the intriguing link between neurodegenerative and oncogenic pathways, revealing a genetic basis for a reduced prostate cancer risk in Alzheimer’s patients. Using bidirectional Mendelian randomization, their study suggests an inverse relationship between neurodegenerative and cancer processes. In the realm of metabolism and carcinogenesis, Wu et al. identify a causal association between blood metabolites and basal cell carcinoma (BCC), with polyunsaturated fatty acids (PUFAs) emerging as significant risk factors. This underscores the potential role of metabolic screening in assessing BCC risk. Additionally, Ding et al. explore the role of inflammatory markers in lung adenocarcinoma through Mendelian randomization, revealing how IL-17A and specific metabolites influence cancer risk. These findings offer valuable insights for anti-inflammatory therapeutic strategies and deepen our understanding of cancer pathogenesis.

We are pleased to note that certain studies within this Research Topic have already garnered multiple citations shortly after publication, underscoring their pivotal impact and relevance across various scientific fields. This citation pattern highlights how these findings are shaping current research directions and informing future investigations, particularly in the interconnected areas of aging, immunology, and metabolism.

Collectively, these articles underscore the critical need for interdisciplinary approaches to drive advancements in endocrine-related cancer research. By integrating perspectives from aging, immunology, and metabolic science, this Research Topic lays a foundation for precision medicine, fostering innovations that address the intricate biological landscapes of endocrine-related cancers.

